# Circulating tumor DNA guided adjuvant chemotherapy in stage II colon cancer (MEDOCC-CrEATE): study protocol for a trial within a cohort study

**DOI:** 10.1186/s12885-020-07252-y

**Published:** 2020-08-20

**Authors:** S. J. Schraa, K. L. van Rooijen, D. E. W. van der Kruijssen, C. Rubio Alarcón, J. Phallen, M. Sausen, J. Simmons, V. M. H. Coupé, W. M. U. van Grevenstein, S. Elias, H. M. Verkooijen, M. M. Laclé, L. J. W. Bosch, D. van den Broek, G. A. Meijer, V. E. Velculescu, R. J. A. Fijneman, G. R. Vink, M. Koopman, And on behalf of the PLCRC-MEDOCC group, Mich S. Dunker, Martijn F. Lutke Holzik, Ronald Hoekstra, Dirkje W. Sommeijer, Jarmila D. W. van der Bilt, Esther C. J. Consten, Geert A. Cirkel, Thijs A. Burghgraef, Emma M. van der Schans, Peter Nieboer, Ron C. Rietbroek, Jan Willem T. Dekker, Arjan J. Verschoor, Koen A. K. Talsma, Rebecca P. M. Brosens, Helgi H. Helgason, Andreas W. K. S. Marinelli, Ignace H. J. T. de Hingh, Corina N. Oldenhuis, Jan Jansen, Henk K. van Halteren, Hein B. A. C. Stockmann, Aart Beeker, Koop Bosscha, Hans F. M. Pruijt, Leontine E. A. M. M. Spierings, Liselot B. J. Valkenburg-Van Iersel, Wouter J. Vles, Felix E. de Jongh, Hester van Cruijsen, Joost T. Heikens, David D. E. Zimmerman, Robert J. van Alphen, Anandi H. W. Schiphorst, Lobke L. van Leeuwen-Snoeks, Jeroen F. J. Vogelaar, Natascha A. J. B. Peters

**Affiliations:** 1 Department of Medical Oncology, University Medical Center Utrecht, Utrecht University, Heidelberglaan 100, 3584 CX Utrecht, The Netherlands; 2 Department of Pathology, Netherlands Cancer Institute, Plesmanlaan 121, 1066 CX Amsterdam, The Netherlands; 3 The Sidney Kimmel Comprehensive Cancer Center, Johns Hopkins University School of Medicine, Baltimore, MD 21287 USA; 4 Personal Genome Diagnostics, Baltimore, MD 21224 USA; 5 Department of Epidemiology and Biostatistics, Amsterdam University Medical Centers, De Boelelaan 1117, 1081 HV Amsterdam, The Netherlands; 6 Department of Surgical Oncology, University Medical Center Utrecht, Utrecht University, Heidelberglaan 100, 3584 CX Utrecht, The Netherlands; 7 Julius Center for Health Sciences and Primary Care, University Medical Center Utrecht, Utrecht University, Heidelberglaan 100, 3584 CX Utrecht, The Netherlands; 8 Department of Pathology, University Medical Center Utrecht, Utrecht University, Heidelberglaan 100, 3584 CX Utrecht, The Netherlands; 9 Department of Laboratory Medicine, Netherlands Cancer Institute, Plesmanlaan 121, 1066 CX Amsterdam, The Netherlands

**Keywords:** Colon cancer, Circulating tumor DNA, ctDNA, Adjuvant chemotherapy, TwiCs

## Abstract

**Background:**

Accurate detection of patients with minimal residual disease (MRD) after surgery for stage II colon cancer (CC) remains an urgent unmet clinical need to improve selection of patients who might benefit form adjuvant chemotherapy (ACT). Presence of circulating tumor DNA (ctDNA) is indicative for MRD and has high predictive value for recurrent disease. The MEDOCC-CrEATE trial investigates how many stage II CC patients with detectable ctDNA after surgery will accept ACT and whether ACT reduces the risk of recurrence in these patients.

**Methods/design:**

MEDOCC-CrEATE follows the ‘trial within cohorts’ (TwiCs) design. Patients with colorectal cancer (CRC) are included in the Prospective Dutch ColoRectal Cancer cohort (PLCRC) and give informed consent for collection of clinical data, tissue and blood samples, and consent for future randomization. MEDOCC-CrEATE is a subcohort within PLCRC consisting of 1320 stage II CC patients without indication for ACT according to current guidelines, who are randomized 1:1 into an experimental and a control arm.

In the experimental arm, post-surgery blood samples and tissue are analyzed for tissue-informed detection of plasma ctDNA, using the PGDx elio™ platform. Patients with detectable ctDNA will be offered ACT consisting of 8 cycles of capecitabine plus oxaliplatin while patients without detectable ctDNA and patients in the control group will standard follow-up according to guideline.

The primary endpoint is the proportion of patients receiving ACT when ctDNA is detectable after resection. The main secondary outcome is 2-year recurrence rate (RR), but also includes 5-year RR, disease free survival, overall survival, time to recurrence, quality of life and cost-effectiveness. Data will be analyzed by intention to treat.

**Discussion:**

The MEDOCC-CrEATE trial will provide insight into the willingness of stage II CC patients to be treated with ACT guided by ctDNA biomarker testing and whether ACT will prevent recurrences in a high-risk population. Use of the TwiCs design provides the opportunity to randomize patients before ctDNA measurement, avoiding ethical dilemmas of ctDNA status disclosure in the control group.

**Trial registration:**

Netherlands Trial Register: NL6281/NTR6455. Registered 18 May 2017, https://www.trialregister.nl/trial/6281

## Background

In patients with stage II colon cancer (CC) the recurrence rate (RR) after surgery is approximately 15–20% [1]. Disease management after surgical resection in stage II CC is still under debate, because the overall survival (OS) benefit of adjuvant chemotherapy (ACT) in this group of patients varies between 2 and 5% only [2, 3]. Moreover, offering ACT in a low-risk population induces an important amount of overtreatment with unnecessary, but sometimes severe toxicity, and costs.

Several prognostic characteristics of stage II CC have been identified to provide better selection of patients that might benefit from ACT. Patients with presence of at least one of the following characteristics are classified as being at high risk of disease recurrence: poorly differentiated histology, pT4 lesions, inadequately (less than 12) sampled lymph nodes, lymphovascular or perineural invasion or tumor presentation with perforation or obstruction [4].

In contrast, patients with a deficient mismatch repair (dMMR) status in stage II CC have a low risk of recurrence and ACT is not considered beneficial, irrespective of the presence of other risk factors [5, 6]. Other known prognostic factors in CC, like gene expression profiles or BRAF (V600E) and RAS mutations, have been investigated but do not adequately identify the patients that will benefit from ACT [7–9].

Despite the definition of high- and low risk subgroups of stage II CC patients, retrospective analyses demonstrated that improved survival after administration of ACT was not observed in high risk patients, or exclusively in patients with a pT4 tumor [10–12]. Therefore in the Netherlands, ACT is currently only recommended in stage II CC patients with a pT4 tumor without dMMR.

Unfortunately, also pT4 is not an absolute predictor for disease recurrence in stage II patients. In a retrospective analysis of 995 stage II CC patients with pT4 tumors, the 3-year disease-specific survival rate after surgery was 91% in patients who received ACT and 73% in patients who did not receive ACT, which means that 73% of these patients are exposed to ACT unnecessarily [12]. Considering non-pT4 stage II patients, a population registry analysis of 40,338 patients showed that in this group 12.5% of patients suffered from recurrences [13]. These data demonstrate that using pT4 as a prognostic factor results in significant under- and overtreatment.

Minimal residual disease (MRD) is defined as the presence of tumor cells in the blood, bone marrow or lymph nodes not detected by conventional staging procedures [14]. Patients who have MRD after surgery are not completely cured and therefore at high risk of developing disease recurrence. Development of a highly specific and sensitive (bio)marker test indicative for MRD would allow identification of the subset of patients likely to experience recurrence of disease, thereby improving the selection of patients who may benefit from adjuvant treatment. In adjuvant trials, this would solve problems of high numbers needed for inclusion and dilution of effectiveness of adjuvant treatment by inclusion of many already cured participants [15].

Cell-free circulating tumor DNA (ctDNA) has a strong potential for being this sensitive, and yet specific biomarker. ctDNA consists of small fragments (usually 150–200 bp) of tumor-derived DNA containing tumor-specific mutations which can be detected in liquid biopsies such as blood samples [16–18]. Because of the short half-life of ctDNA (estimates ranging from 15 to 120 min) the presence of ctDNA in blood samples taken several days after surgery presumably reflects a state of MRD [19–21]. Patients with MRD have the highest risk for disease recurrence.

Recently, the presence of ctDNA after tumor resection demonstrated a very strong prognostic value for disease recurrence in stage II CC, with a 2-years RR of 79.0% versus 9.8% in patients with and without detectable ctDNA after surgery respectively [21]. In this study the univariate prognostic value of ctDNA was much higher than that of pT4 status (hazard ratio of 14 versus 2.6, respectively). There are several ongoing trials that use ctDNA in prognostication (NCT03637686, NCT03737539, NCT03416478, NCT03312374, NCT02842203, NCT03615170) and treatment (NCT03748680, ACTRN12615000381583, NRG-GI 005) of non-metastatic CC, but to date there are no results available of randomized controlled trials (RCTs) that use ctDNA for selection of ACT treatment.

The accumulating evidence for the strong prognostic value of ctDNA raises an important ethical dilemma for randomization of patients when designing a conventional RCT, in which patients with detectable ctDNA are randomized into ACT treatment or standard of care follow-up while disclosing ctDNA status to the control group. Indeed, the knowledge of having a very high chance of disease recurrence will be a big burden for patients with detectable ctDNA in the control group and their caregivers as they are not being offered any additional therapy. This warrants an innovative trial design different from the conventional RCT, like the ‘Trial within Cohorts’ (TwiCs) design [22–25]. The TwiCs design enables an experimental group in which ctDNA status is disclosed and a control group that is unaware of their ctDNA status.

The MEDOCC-CrEATE trial is designed as a multicenter TwiCs study with two parallel groups in which we will investigate whether stage II CC patients with detectable ctDNA after resection are willing to receive ACT and whether ACT reduces the RR in these ctDNA-positive patients.

## Methods/design

### Aim

This study investigates the willingness of patients to receive ACT after detection of ctDNA post-surgery and the effect of ctDNA-guided ACT on the RR in stage II CC patients.

### Study design

**Fig. 1:**
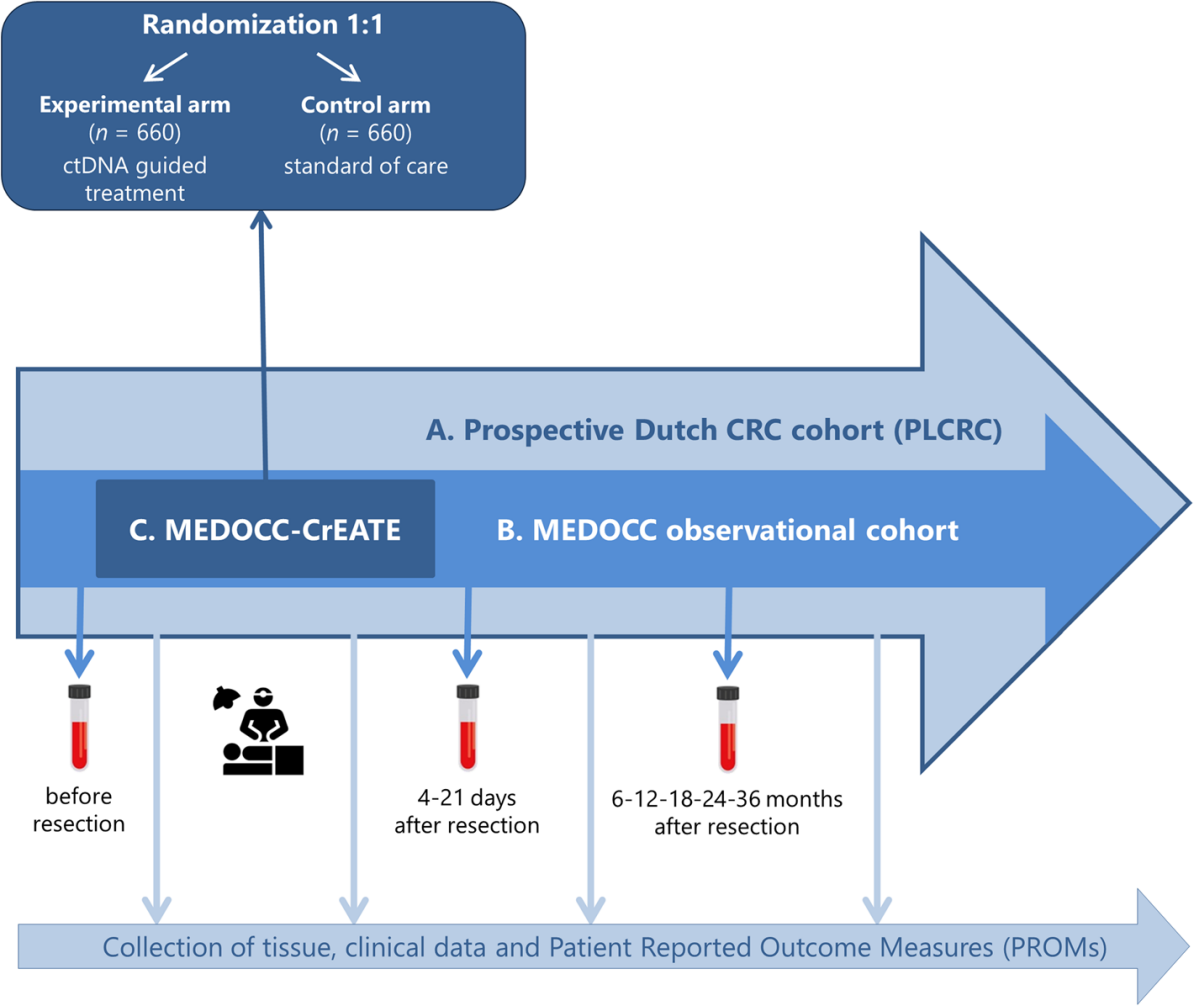
Schematic presentation of MEDOCC-CrEATE, using the trial within cohort (TwiCs) design. **a** PLCRC is a nationwide cohort study in the Netherlands with inclusion of CRC patients (all stages). By optional informed consent regarding collection of biomaterials and future randomization, observational as well as interventional trials can be performed within the cohort. **b** Non-metastatic CRC patients are included in MEDOCC when the patient signs informed consent for PLCRC including additional blood sampling. Blood samples are withdrawn before resection, 4–21 days after resection and every 6 months during the first 3 years of follow-up. **c** Eligible stage II colon cancer patients are randomized 1:1 following the TwiCs design. In the experimental group informed consent is being asked for immediate ctDNA analysis of the blood sample obtained after resection. If ctDNA is detectable, patients are offered adjuvant chemotherapy. The control group is not informed about MEDOCC-CrEATE and will receive standard of care

The MEDOCC-CrEATE trial follows the TwiCs design and is performed within the Prospective Dutch ColoRectal Cancer cohort (PLCRC; www.PLCRC.nl) [26]. PLCRC is set up by the Dutch Colorectal Cancer Group (DCCG) and collects clinical data and Patient Reported Outcome Measures (PROMs) at baseline and at multiple time points during follow-up (Fig. 1). At enrollment, patients give informed consent for use of their clinical data and optionally for receiving quality of life questionnaires, collection of biomaterials for research, additional sequential blood sampling and for being approached for future studies conducted within the infrastructure of the cohort, either in accordance with the TwiCs design or not.

### Patient selection and recruitment

Patients will be recruited in both academic and non-academic hospitals in the Netherlands that are participating in PLCRC. Non-metastatic colorectal cancer (CRC) patients that give informed consent for PLCRC including consent for additional blood sampling at enrollment, will be included in the observational PLCRC substudy MEDOCC (Molecular Early Detection of Colon Cancer) before surgery. The participants are eligible for the current MEDOCC-CrEATE trial if they meet the following criteria after surgery: (1) histopathological confirmed and radically resected stage II CC; (2) age ≥ 18 years; (3) informed consent for PLCRC and MEDOCC including consent for randomization in future trials and use of tissue; (4) physical condition allows treatment with combination chemotherapy consisting of a fluoropyrimidine and oxaliplatin; and (5) no indication for ACT according to the treating physician and/or multidisciplinary board. Patients who are pregnant, have had another malignancy in the previous 5 years, except for carcinoma in situ, or patients with contra-indications for fluoropyrimidines and/or oxaliplatin will be excluded.

Currently the Dutch guidelines recommend ACT for patients with pT4 tumors. However, there is large age- and hospital dependent variation in administration of ACT in this group and in clinical practice not all stage II patients with pT4 tumors will be offered ACT [27]. Therefore, we will include eligible patients with pT4 tumors without a recommendation for ACT according to their treating physician and use pT4 status as a stratification factor.

### Blood sample collection

Blood samples are collected before and 4–21 days after surgery for all patients included in the MEDOCC clinical study, predominantly comprising stage I, II and III CC patients (Table 1). Blood samples (two tubes of 10 ml per timepoint) are collected in Cell free DNA Streck Blood Collection Tubes for various research purposes, among which the MEDOCC-CrEATE trial.

**Table 1:**
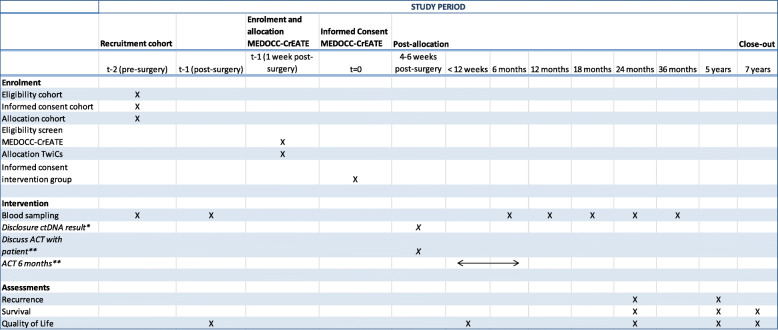
Standard Protocol Items for Intervention Trials (SPIRIT): schedule of enrollment, interventions and repeated measurements. ACT: adjuvant chemotherapy; ctDNA: circulating tumor DNA; QoL: quality of life. * Intervention group only. ** Intervention group only, if ctDNA is positive

### Randomization

About 1 week after surgery, when the histopathological report is finished, MEDOCC patients who are eligible for MEDOCC-CrEATE will be randomized 1:1 to the intervention or control arm using SLIM, an online platform to manage patient inclusion including a randomization service. The computer generated randomization schedule is stratified by T-stage and uses permuted blocks of random sizes. Allocation concealment will be ensured, as the service will not release the randomization code. Only patients randomized to the intervention arm will be informed about MEDOCC-CrEATE according to the TwiCs design [22].

### Experimental arm

After randomization, only patients randomized to the experimental arm will be asked separate informed consent for the immediate analysis of ctDNA status of the post-surgery sample. A small proportion of patients, estimated approximately 5–8% will have detectable ctDNA in their blood. These patients will be offered ACT. Patients decide whether they accept or refuse this treatment. Patients without detectable ctDNA will receive routine standard of care.

ACT will consist of 6 months of capecitabine and oxaliplatin (CAPOX) or 6 months of fluorouracil, leucovorin and oxaliplatin (FOLFOX). Treatment starts preferably within 8 weeks and not beyond 12 weeks after surgery.

During and after completing ACT routine follow-up will consist of regular visits at the surgical outpatient department, blood withdrawals for analysis of carcinoembryonic antigen (CEA) and imaging (standard ultrasound of the liver) according to current guidelines in the Netherlands. No additional imaging will be performed to prevent detection bias.

### Control arm

In the control arm, patients will not be informed about the MEDOCC-CrEATE trial and receive routine follow-up care consisting of CEA tests every 3 months for the first 3 years and abdominal ultrasound or CT every 6 months in the first year and once a year afterwards. One year after surgery a colonoscopy is performed. Post-surgery blood samples will not be tested for ctDNA immediately, but will be analyzed batch-wise after several months without result disclosure to patients and their treating physicians.

### Follow-up

Blood samples will be collected at 6-monthly intervals for the first 3 years after surgery for both patients in the experimental arm and the control arm conform the MEDOCC study protocol. These samples will not be analyzed for ctDNA immediately and results will not be disclosed to patients and treating physicians.

### Tumor tissue-informed ctDNA analysis

After surgery the local pathologist will send a formalin-fixed paraffin-embedded (FFPE) tissue block to the central laboratory, where DNA will be isolated for further analysis.

The post-surgery blood sample is drawn between 4 and 21 days after surgery. The sample is not withdrawn before day 4 to reduce the risk of false-negative ctDNA tests due to the relatively large amount of cell free DNA (cfDNA) released due to cell damage after surgery. The blood is taken no later than 21 days after surgery to be able to start chemotherapy within 12 weeks after surgery. Samples are kept at room temperature and sent by regular mail to the central laboratory within 1–2 days, where ctDNA will be isolated for further analysis.

Tumor tissue DNA will be analyzed by targeted next generation sequencing of a panel of more than 500 genes using the PGDx elio™ tissue complete assay from Personal Genome Diagnostics (PGDx, Baltimore, MD, USA). Plasma ctDNA will be analyzed by targeted next generation sequencing of a panel of more than 30 genes using the PGDx elio™ plasma resolve assay from PGDx (Baltimore, MD, USA). Both panels include the most commonly mutated genes in CC, including *APC*, *TP53*, *KRAS* and *BRAF*. Tumor tissue DNA mutations are used as input information for plasma ctDNA mutation calling, thereby increasing both sensitivity and specificity of the ctDNA test.

### Primary endpoint

The primary endpoint is the proportion of patients starting with ACT after detection of ctDNA in the post-surgery sample.

### Secondary endpoints

The most important secondary endpoint is 2-year RR in patients with detectable ctDNA in their blood, expressed as the proportion of patients that experience a recurrence within 2 years after surgery. Detection of recurrences (in months after surgery) will occur by standard follow-up investigations including 3–6 monthly blood sampling of tumor marker CEA and 6 monthly imaging with ultrasound liver or CT abdomen and when indicated by symptoms. Radiological and/or histopathological evidence is used to confirm the recurrence; the date of the said investigation is considered the date of recurrence.

Data about follow-up, recurrences and survival are routinely collected within PLCRC using the Netherlands Cancer Registry (NCR), managed by the Netherlands Comprehensive Cancer Organisation (IKNL) to provide insight in the characteristics and magnitude of cancer in the Netherlands [28].

Other secondary endpoints include 2-year RR in a per-protocol analysis, 5-year RR (intention-to-treat and per-protocol analysis), time to recurrence (TTR), 2- and 5-year disease free survival (DFS) rate, 5- and 7-year disease-related OS rate, 2- and 5-year RR in patients with undetectable ctDNA after surgery, quality of life (QoL) and cost-effectiveness of the ctDNA-guided strategy.

#### Time-to-event outcomes

OS rate is expressed as proportion of patients that are alive 5 and 7 years after surgery. DFS rate is expressed as proportion of patients that did not experience disease recurrence, a second primary CC or death within 2 and 5 years after surgery. TTR is expressed as time (months) between surgery and detection of disease recurrence. Patients will be censored at the last date of follow-up if a date of death is not recorded and at the date of death if the cause of death is not due to CC.

#### Quality of life

QoL is measured within the cohort at regular intervals in patients who gave consent to send questionnaires. Nationally and internationally validated questionnaires are used, among which the European Organisation for Research and Treatment of Cancer Quality of Life Questionnaire Core 30 and the ColoRectal cancer module (EORTC-QLQ-C30 and -CR29), the Work Ability Index (WAI), the Euro Quality of life-5 Dimensions (EQ-5D), the Multidimensional Fatigue Inventory-20 (MFI-20) and the Hospital Anxiety and Depression Score (HADS).

#### Cost-effectiveness of the ctDNA-guided treatment

The cost-effectiveness analysis will be carried out from a societal perspective, including both direct health care costs as well as indirect costs from productivity loss. The health outcome measure in the cost-effectiveness analysis will be the total quality adjusted life years (QALY) per group. For analysis of factors related to QALYs questionnaires are used, provided within PLCRC.

### Sample size considerations

The primary endpoint is the proportion of ctDNA positive patients starting with ACT. However, 2-year RR in the ctDNA positive patients after surgery is an important secondary endpoint and the power calculation is performed for this secondary endpoint. We estimate that, similar to effectiveness in stage III CC patients, ACT in ctDNA-based high-risk stage II CC patients will lead to a 30% absolute reduction of recurrences within 2 years after surgery. In the observational trial 79% of patients with detectable ctDNA experienced disease recurrence within 2 years after resection [21].

With a power of 80% and an alpha of 0.05, 30 patients with detectable ctDNA need to be included in both arms. Assuming a prevalence of ctDNA after surgery of 5%, and adjustment for loss to follow-up and rejection of adjuvant therapy in the intervention arm of 10%, a total sample size of 1320 patients is calculated (660 in each arm). We expect few patients with detectable ctDNA in the intervention group to refuse ACT, because patients are selected upfront for being in a physical condition to receive ACT and the established prognostic value of detectable ctDNA is high.

We assume that cross-over from the control arm to the intervention arm will not occur, because only eligible patients randomly selected in the cohort and allocated to the intervention arm will be informed about the trial and have the opportunity for immediate analysis of ctDNA. Patients in the control group will not be informed about the trial or their ctDNA status.

We assume that 90% of patients in the intervention arm with detectable ctDNA will be treated with ACT. The proportion of patients starting with chemotherapy, the primary endpoint, can in that instance be determined with a margin of error (width of the 95% confidence interval) of 11%.

We expect to complete recruitment of patients within 2–3 years with more than 20 participating Dutch hospitals.

### Data analysis

Data will be analyzed according to the intention-to-treat principle for the primary endpoint and the secondary endpoint of 2-year RR in patients with detectable ctDNA after surgery. In this analysis we expect to compare 30 patients with detectable ctDNA who received ACT in the intervention arm with 30 patients with detectable ctDNA in the control arm, i.e. based on ctDNA analysis performed retrospectively, at least 3 months after surgery, and not disclosed to patients and treating physicians. The proportion of patients that experience a recurrence in both arms will be compared by means of a chi-square test. In addition, for other secondary endpoints and exploratory analyses we will analyze time-to-event outcomes in patients in both arms with detectable ctDNA after surgery. Differences in time-to-event outcomes will be analyzed by standard survival methods, e.g. Kaplan-Meier curves compared by log-rank tests. Cox’s proportional hazards models will be used for multivariable analysis.

Comparison of QoL of the ctDNA positive patients in both study arms will be done using repeated measurements methods and including ACT as factor. QoL will also be analyzed for the whole population in both arms of the study. Treatment differences at each QoL assessment time point will be compared by means of the Wilcoxon Rank Sum Test.

A lifetime horizon will be applied for the cost-effectiveness analysis, parametric survival functions will be used to extrapolate DFS and OS curves beyond 5 years.

### Responsibilities

Protocol modifications will be submitted as amendment to the medical ethical committee by the study coordinator. The local principle investigator of each participating hospital is responsible for patient inclusion, logistics of biomaterials to the central laboratory and patient follow-up. To ensure quality of data, study integrity and compliance with the protocol and the various applicable regulations and guidelines, a data monitor of the IKNL has been appointed to conduct site visits to the participating centers and randomly check patient data. The study coordinator – together with the principle investigator - will have access to the final dataset and is responsible for publishing study results. The results will be submitted to a peer-reviewed journal.

## Discussion

MEDOCC-CrEATE is the first clinical trial using the TwiCs design to investigate ctDNA-guided strategies in stage II CC, taking an important step towards clinical implementation of ctDNA in cancer diagnostics and care.

A few other trials with the aim to reduce recurrences in CC by use of a ctDNA-guided approach are in preparation or recently started. The IMPROVE-IT trial, a Danish study started in October 2018, uses a classical RCT in stage I and II CRC patients, randomizing between 6 months of ACT or intensified follow-up for 64 patients with detectable ctDNA post-surgery (NCT03748680). Four hundred fifty stage II CRC patients are being included in the Australian DYNAMIC study and randomized 2:1 to be treated according to the ctDNA result with 3 to 6 months of ACT or according to standard of care (ACTRN12615000381583). The COBRA study in the United States and Canada has a similar RCT approach (NRG-GI 005). Also, several trials in stage III CRC patients started recently (DYNAMIC III, ACTRN12617001566325). In the near future these studies will provide deeper understanding and lead to implementation of ctDNA-guided strategies in clinical practice.

In the current era of rapidly emerging new diagnostic and treatment strategies, the classical RCT is challenged because of inefficient and therefore time-consuming recruitment of eligible patients. Main reasons for patients to refrain from participation in RCTs are preference for one of the treatment arms, anxiety or aversion to randomization and difficulties understanding the concept of an RCT, resulting in a delay of availability of potential beneficial treatments [29]. Modern trial designs are being adopted to avoid this inefficient, time-consuming and costly way of conducting trials with high rates of unfinished studies. Therefore, the MEDOCC-CrEATE trial uses the modern TwiCs design. The TwiCs design has shown to have a positive impact on trial efficiency. Also, by enrolling higher proportions of eligible patients generalizability to daily clinical practice improves [25].

This study design has several strengths. First, MEDOCC-CrEATE is nested within the large nationwide PLCRC cohort study with currently almost 8000 included CRC patients. The infrastructure of this cohort, in which clinical data and biomaterials are collected after broad informed consent of participating patients, allows comprehensive, innovative and efficient research in CRC. Using this infrastructure, the study can be quickly implemented in many participating hospitals, saving costs and complicated logistics. Several studies according to the TwiCs design are performed within this or comparable cohorts. Therefore experience with this trial design has been gained and this will contribute to execution of the MEDOCC-CrEATE study [30, 31].

Secondly, a difficult ethical dilemma in an RCT analyzing ctDNA presence post-surgery is avoided by the TwiCs design. With the current knowledge about the strong association with recurrent disease, disclosing ctDNA status to all participants would be a great burden for patients with detectable ctDNA and their treating physicians in the control group. Because of ‘disappointment bias’ in the control group we would expect high drop-out and contamination due to cross-over when a classical RCT design would be applied, making accrual and interpretation of results unfeasible [32]. In this TwiCs study, all participants already have blood withdrawn after surgery for research purposes, and only the eligible patients allocated to the intervention arm will have the opportunity to obtain a ctDNA test result and ACT if ctDNA is detected. Patients in the control arm, treated according to current guidelines, will not be informed about randomization and their blood samples will be analyzed at a later point in time beyond the window of ACT treatment.

This study has also potential limitations and challenges. The TwiCs design is potentially susceptible to low statistical power and internal validity biases. Levels of participant’s eligibility and consent should be substantial to achieve valid and reliable results, and measurements taken in the control group should be sufficient for adequate comparisons to be made [33]. Therefore the TwiCs design is not appropriate for every experimental intervention. In case of the MEDOCC-CrEATE study, we argue that eligibility and also consent will be substantial because of the high incidence of CC, the large cohort with high inclusion rates and the assumption that eligible patients in the intervention group are willing to accept ACT because of the very strong association of the presence of ctDNA with recurrent disease.

Another limitation is the small sample size for primary outcome analysis. Eventually only 30 patients in both arms of the trial are expected to have detectable ctDNA after surgery. Based on previous data, 80% relapses are expected within 2 years, and with a high event rate small numbers are sufficient [21].

We recommend a 6-month duration of ACT consisting of capecitabine and oxaliplatin (CAPOX) or fluorouracil, leucovorin and oxaliplatin (FOLFOX) for patients with detectable ctDNA after surgery. The first adjuvant CC trials investigating the combination of a fluoropyrimidine and oxaliplatin reported results for 6 month duration of ACT [34]. In 2018 the IDEA trial found a large reduction in toxicity for 3 months treatment compared to 6 months treatment. Although this trial could not confirm non-inferiority for 3 months treatment for all patients treated with CAPOX or FOLFOX in stage III CRC, the small difference limits clinical relevance. Besides, it did show non-inferiority of the shorter regimen in patients treated with CAPOX. Consequently, Dutch guidelines recommend 3 months of ACT for CC since 2019. However, among patients with highest risk of recurrence (T4, N2, or both) superiority of 6-month duration of therapy was found. Additional IDEA-FRANCE results, presented at the ESMO Congress 2019, showed the worst prognosis for ctDNA positive patients who only received 3 months of ACT [35]. Therefore in this study, we recommend 6-months ACT for patients with a very high risk of disease recurrence due to the presence of ctDNA after surgery.

Liquid biopsy ctDNA detection has become a promising technology with multiple putative clinical applications, including its potential use as a biomarker for early diagnosis, prognosis, prediction, and monitoring of treatment response [36]. Driven by the excitement of its possibilities, the field of technology of ctDNA detection and analysis is rapidly evolving. Yet, the clinical utility of ctDNA testing still needs to be proven. When to apply what technology to address which unmet clinical need is a key question that remains to be addressed [18].

Applying ctDNA detection as a biomarker for MRD is a challenging task. Biologically, only a very low amount of ctDNA is present in post-surgery patients with MRD. Stochastically, by looking at mutations in a panel of genes chances increase that in a given blood sample at least in one of the genes a mutation can be reliably detected. Test sensitivity can be further increased by making use of DNA mutation information from tumor tissue, because the stringency in the calling of plasma ctDNA mutations can be reduced once you know what mutations to look for. Tissue-informed ctDNA analysis also increases the ctDNA test specificity. Recent observations showed that ctDNA mutation detection can be confounded by mutations that are present in clonal hematopoiesis, including mutations in genes that are commonly affected in CC such as *TP53* [37]. These confounding mutations can be filtered by applying tissue-informed ctDNA analyses. As such, technically the MEDOCC-CrEATE trial makes use of a ctDNA test that is well-suited for MRD detection [38]. Clinically, however, the MEDOCC-CrEATE trial needs to resolve whether a positive ctDNA test also allows to select for patients who truly benefit from ACT treatment, a requirement for clinical implementation. To further support clinical implementation of ctDNA analyses in the Netherlands, the Dutch COIN initiative aims to provide a validation framework for clinical implementation of ctDNA analyses in the Netherlands (ZonMW project number 848101011).

In conclusion, the MEDOCC-CrEATE study is the first study using the modern and innovative TwiCs design to study ctDNA-guided administration of ACT in stage II CC patients. The study aims to answer the important clinical question whether ctDNA has prognostic as well as predictive value. If this study demonstrates a significant and substantial difference in disease recurrence in the intervention group compared to the control group, ctDNA analysis and ctDNA-guided treatment should be implemented into clinical practice to improve the prognosis of stage II CC patients.

## Data Availability

Not applicable.

## Abbreviations

ACT: Adjuvant ChemoTherapy
CC: Colon Cancer
CEA: CarcinoEmbryonic Antigen
CRC: ColoRectal Cancer
ctDNA: Circulating tumor DNA
DFS: Disease Free Survival
dMMR: Deficient MisMatch Repair
EORTC-QLQ-C30 and -CR29: European Organisation for Research and Treatment of Cancer Quality of Life Questionnaire C30 and Colorectal cancer module 29
EQ-5D: Euro Quality of life-5 Dimensions
FFPE: Formalin-Fixed Paraffin-Embedded
HADS: Hospital Anxiety and Depression Score
IKNL: Netherlands Comprehensive Cancer Organisation
MFI-20: Multidimensional Fatigue Inventory-20
MRD: Minimal Residual Disease
NCR: Netherlands Cancer Registry
OS: Overall Survival
PLCRC: Prospective Dutch ColoRectal Cancer cohort
PROMs: Patient Reported Outcome Measures
QALY: Quality Adjusted Life Year
QoL: Quality of Life
RCT: Randomized Controlled Trials
RR: Risk of Recurrence
TTR: Time To Recurrence
TwiCs: Trial within Cohorts study
WAI: Work Ability Index
